# Liver transplant after SARS-CoV-2 infection: A systematic review

**DOI:** 10.1016/j.clinsp.2022.100042

**Published:** 2022-04-26

**Authors:** Lucas S. Nacif, Michel Ribeiro Fernandes, Daniel R. Waisberg, Rafael S. Pinheiro, Vinicius Rocha-Santos, Flávio Galvão, Wellington Andraus, Luiz Carneiro-D'Albuquerque

**Affiliations:** Liver and Gastrointestinal Transplant Division, Department of Gastroenterology, Faculdade de Medicina, Universidade de São Paulo, São Paulo, SP, Brazil

**Keywords:** Liver transplantation, Systematic review, COVID-19, Humans, Liver diseases, SARS-CoV-2, 2019-nCoV, Solid-organ transplant recipient, COVID-19, Coronavirus Disease 2019, LT, Liver Transplantation, PRISMA, Preferred Reporting Items for Systematic reviews and Meta-Analysis, PROSPERO, Prospectively Registered Systematic Reviews, SOT, Solid Organ Transplant, SARS-CoV-2, Severe Acute Respiratory Syndrome Coronavirus-2

## Abstract

**Background:**

The Coronavirus 19 (COVID-19) pandemic has dramatically impacted liver organ transplantation. The American Society of Transplantation recommends a minimum of 28 days after symptom resolution for organ donation. However, the exact time for transplantation for recipients is unknown. Considering that mortality on the waiting list for patients with MELD >25 or fulminant hepatitis is higher than that of COVID-19, the best time for surgery after SARS-CoV-2 infection remains undetermined. This study aims to expand the current knowledge regarding the Liver Transplantation (LT) time for patients after COVID-19 and to provide transplant physicians with essential decision-making tools to manage these critically ill patients during the pandemic.

**Methods:**

Systematic review of patients who underwent liver transplantation after diagnosis of COVID-19. The MEDLINE, PubMed, Cochrane, Lilacs, Embase, and Scielo databases were searched until June 20, 2021. The MESH terms used were “COVID-19” and “Liver transplantation”.

**Results:**

558 articles were found; of these 13 articles and a total of 18 cases of COVID-19 prior to liver transplantation were reported. The mean age was 38.7±14.6, with male prevalence. Most had mild symptoms of COVID. Five patients have specific treatment for COVID-19 with convalescent plasm or remdesivir/oseltamivir, just one patient received hydroxychloroquine, and 12 patients received only symptomatic treatment. The median time between COVID-19 to LT was 19 days (13.5‒44.5). Deceased donor liver transplantation accounted for 61% of cases, while living donor transplantation was 39%.

**Conclusion:**

Despite the concerns regarding the postoperative evolution, the mortality of patients with high MELD or fulminant hepatitis transplanted shortly after COVID-19 diagnosis does not seem to be higher.

(PROSPERO, registration number = CRD42021261790)

## Highlights

• The Coronavirus 19 (COVID-19) pandemic has dramatically impacted liver organ transplantation.
• This study systematically reviewed the current knowledge regarding the Liver Transplantation (LT) time for patients after COVID-19.
• Furthermore, the authors provide more knowledge to the transplant physicians with essential decision-making tools to manage these critically ill patients during the pandemic.

## Introduction

Coronavirus disease 2019 (COVID-19) is caused by a novel coronavirus termed Severe Acute Respiratory Syndrome Coronavirus-2 (SARS-CoV-2).^1^^,^^2^ The disease has spread worldwide and has become a public health emergency pandemic of international concern.^3^

The American Society of Transplantation (AST) recommends procuring an organ from a COVID-19-positive donor only after 28 days following the resolution of all symptoms. However, it is unclear when a recipient should undergo Liver Transplantation (LT) after COVID-19 diagnosis.^4^ Furthermore, the AST guideline recommends at least one negative COVID-19 RT-PCR test from the respiratory tract within three days before transplantation.^5^ Even though it is suggested to avoid postponing LT in patients with a high Model for End-Stage Liver Disease score (MELD >25) or those with acute liver failure, the timing of surgery after SARS-Cov-2 infection in a recipient is not known.^6^ Thus, the best time for transplantation after SARS-CoV-2 infection remains undetermined for these patients.

The authors performed a systematic review of the existing literature to aid in expanding the current limited knowledge regarding the time of LT for patients after COVID-19, mainly focusing on clinical presentations, treatment modalities, and outcomes. The authors aim to provide transplant physicians worldwide with essential decision-making tools to manage these critically ill patients during this time of crisis.

## Methods

### Study identification

A systematic review of the literature on liver transplantation after COVID-19 infection was carried out. The MEDLINE-PubMed, EMBASE, Cochrane Library, LILACS, SciELO, and Web of Science databases were electronically searched and updated until June 20, 2021. The MeSH terms used were “COVID-19” (entire related MeSH terms: 2019 novel coronavirus, SARS-CoV-2 infection, 2019-nCoV infection) AND “Liver transplantation”.

The terms and MeSH terms for databases search were developed with the PICO structure: Patient, Intervention, Comparison or Control, and Outcome (PICO). The terms for each group were combined with the “OR” operator. The results of the search terms forming the “P” (Patients) group were combined with those of search terms forming the “I” (Intervention) group, with “AND”, and for exclusion terms, with “NOT”.

Participants/population: Adults and children who had COVID-19 infection and underwent liver transplantation. Intervention(s), exposure(s): Adults and children who tested positive for COVID-19 that progressed or not to severe acute respiratory syndrome and posteriorly underwent LT. Comparator(s)/control: Patients who were not exposed to SARS-CoV-2 infection before LT. The authors evaluated two LT modalities (DDLT and LDLT) and various epidemiologic groups such as those matched for age, sex, infection interval to LT, and specific treatment for COVID-19. Context Main outcome(s): Survival after SARS-CoV-2 infection and LT.

This systematic review was registered in the international database of prospectively registered systematic reviews (PROSPERO, registration number CRD42021261790). The review protocol can be accessed online via the PROSPERO website (https://www.crd.york.ac.uk/prospero/). The Preferred Reporting Items for Systematic Reviews and Meta-Analysis (PRISMA) checklist was adhered to when preparing this manuscript.^7^, ^8^, ^9^ The review methodology followed the recommendations published by PRISMA.^7^, ^8^, ^9^

### Study selection

Inclusion and exclusion criteria: Selection criteria were used within the research question of the PICO structure. All studies evaluated were written in English.

Case reports, letters to the editor, clinical randomized controlled trials, non-randomized controlled trials, reviews, consensus articles, and protocol studies were included. Studies on organs other than the liver, those on a novel therapy for COVID-19, those on the impact of COVID-19 on the transplant system, those on vaccine research, those involving COVID-19 patients after LT, those on the clinical manifestation of COVID-19 on cirrhosis, epidemiologic studies, those on elective or non-transplant surgical procedures, and those on immunosuppression protocols that were unrelated to LT after COVID-19 infection were excluded.

### Study data extraction

Data extraction was carried out independently by two researchers, using the text, tables, and figures of the original published articles. The quality of the studies selected and the selection methods were evaluated by two independent researchers (MRF and LSN). In the case of a disagreement, the researchers held a consensus meeting to reach a final decision.

### Statistical analysis

Quantitative and qualitative variables were presented as number and percentage, median and range, or mean and standard deviation. A Mann-Whitney *U* test was used to compare independent samples, and p < 0.05 was considered significant. All tests were performed using Graph Prism version 9 (GraphPad Software, Inc, San Diego, CA, USA), with a=0.05 and a 95% Confidence Interval.

## Results

**Fig. 1:**
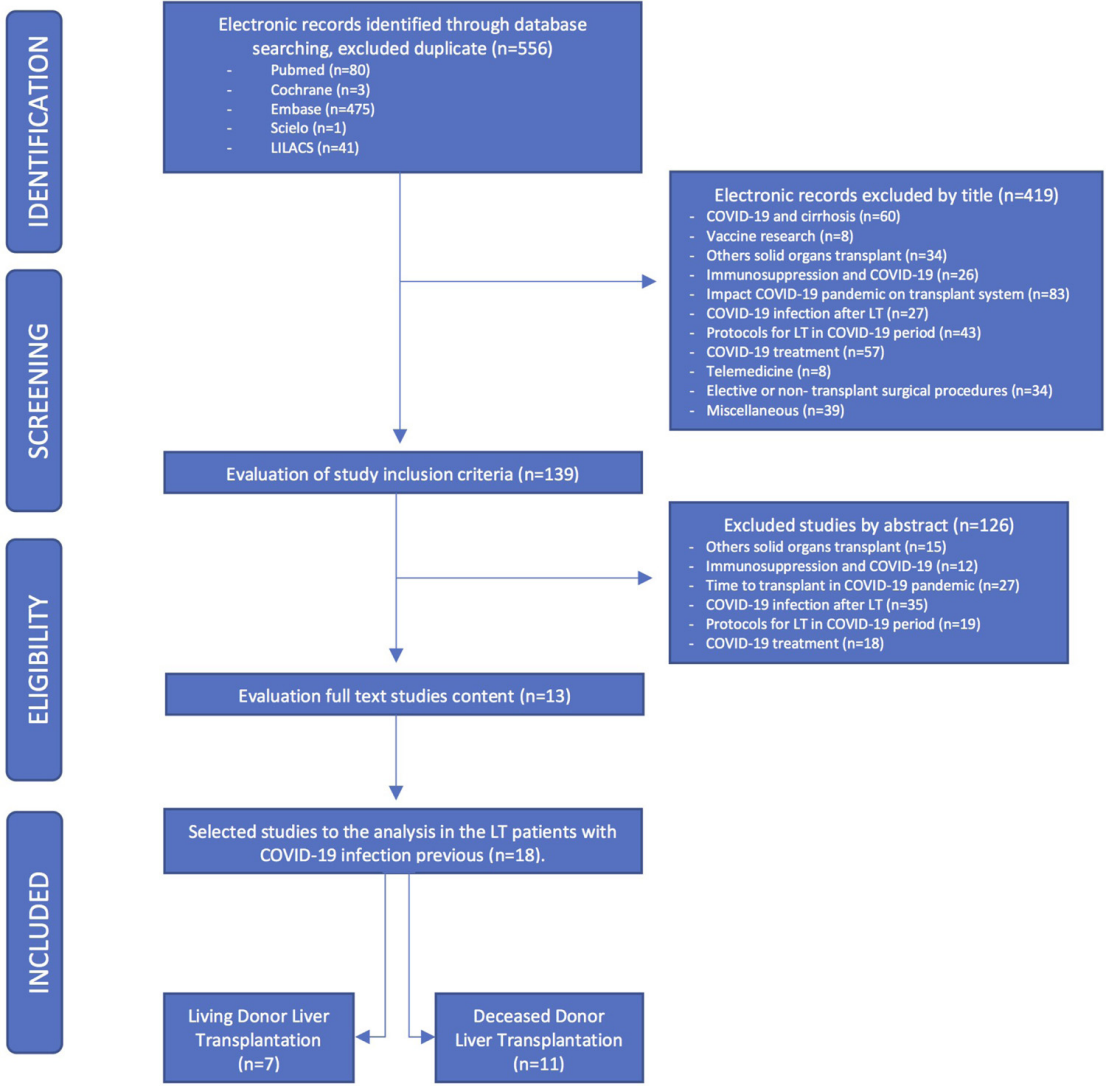
Flow diagram of systematic literature search according to the PRISMA statement. *COVID-19 (2019 novel coronavirus, SARS-CoV-2 infection, 2019-nCoV infection) AND “Liver transplantation”.

The literature search revealed 558 articles, of which 13 articles were selected and analyzed in this review (Fig. 1). The authors did not find well-designed randomized control trials, cohorts, or prospective or retrospective studies. Most articles were case reports, case series, letters to the editor, and editorials.

Among 545 articles were excluded and shown in Fig. 1: 39 about other organs than the liver; 75 about a novel therapy for COVID-19; 83 about the impact of COVID-19 on the transplant system; 8 about vaccine research; 6 about COVID-19 patients after LT; 60 about the clinical manifestation of COVID-19 on cirrhosis; 34 about epidemiologic studies; 52 about COVID-19 protocols; 34 about the elective or non-transplant surgical procedures; and 38 about the immunosuppression protocols that were unrelated to LT after COVID-19 infection.

Data extraction and synthesis were performed using articles addressing patients with COVID-19 who underwent LT. There were 13 articles selected (Table 1).

**Table 1:** Data extraction and synthesis of COVID-19 infection patient who were submitted to LT.

	Age (yr)/ Sex	Etiology of cirrhosis	MELD	Severity of COVID	Specific treatment for COVID	COVID antibodies	Time to LT (days)	Modality	Time to discharge (days)	Immunosuppression	Follow-up	Paper type
Yohanathan, 2021	18/F	Wilson disease	43	Mild	Convalescent Plasm and Remdesivir		17	DDLT		MP, basiliximab and TAC		CR
Tuncer, 2021	60/M	Cryptogenic	19	Mild	None		21	LDLT		MP, TAC and MMF	16 days	CR
Tabrizian, 2020	57/F	HCV		Moderate	None	Detectable	89	DDLT	7	MP, TAC and MMF	5 months	LE
Rouphael, 2020	27/F	FHF (acetoaminophen)		Mild	Convalescent Plasm		4	DDLT	27	MP and TAC		CR
Raut, 2021	36/M	Alcohol		Moderate	None		180	DDLT	10	MP, TAC and MMF		LE
Niess, 2020	56/M	HBV		Mild	None	Detectable	36	DDLT	33	MP and TAC	3.5 months	CR
Kulkarni, 2021	18/M	AIH	29	Mild	None	Non-detectable	17	LDLT		MP, TAC and MMF	60 days	CS
	41/M	Cryptogenic	24	Mild	Remdesivir	Non-detectable	15	LDLT		MP, TAC and MMF	24 days (death)	
	40/F	AIH	29	Moderate	Remdesivir	Detectable	16	LDLT		MP, TAC and MMF	91 days	
	36/M	Alcohol	28	Mild	None	Detectable	16	LDLT		MP, TAC and MMF	91 days	
	37/M	Alcohol	27	Mild	None	Detectable	32	LDLT		MP, TAC and MMF	68 days	
	43/M	NASH	16	Mild	None	Detectable	30	LDLT		MP, TAC and MMF	56 days	
Goss, 2020	4/M	Hepatoblastoma		Mild	None	Detectable	53	DDLT		MP and TAC	42 days	CR
Gambato, 2021	63/F	Alcohol	19	Mild	None	Non-detectable	52	DDLT		MP, TAC and MMF	6 months	LE
Dhand, 2020	42/M	Alcohol	33	Mild	Convalescent Plasm		71	DDLT	25	MP, TAC		CR
Martini, 2020	39/F	AIH	36	Moderate	Hydroxychloroquine	Non-detectable	9	DDLT	9	MP, basiliximab, TAC and MMF		LE
Gao, 2020	37/M	HCV and HCC		Mild	Oseltamivir		3	DDLT	60	MP and TAC	60 days	LE
Manzia, 2021	33/F	HBV	32	Mild	None	Detectable	9	DDLT		MP, TAC and EVE	60 days	CR

COVID, Coronavirus Disease; LT, Liver Transplantation; HCV, Hepatitis C Virus; HBV, Hepatitis B Virus; FHF, Fulminant Hepatitis Failure; AIH, Autoimmune Hepatitis; NASH, Non-Alcoholic Steatohepatitis; HCC, Hepatocellular Carcinoma; MP, Methylprednisolone; TAC, Tacrolimus; MMP, Mycophenolate Mofetil; EVE, Everolimus; DDLT, Deceased Donor Liver Transplantation; CR, Living donor liver transplantation; MELD, Model for End-staged Liver Disease; CR, Case Report; CR, Case Report; CR, Case Report; LE, Letter to Editor; CS, Case Series.

Eighteen patients who underwent LT after COVID-19 were reported. These cases involved transplants in pediatric and adult patients. The mean age was 38.7 ± 14.6 years old with male prevalence, and the majority had mild symptoms of COVID-19. Five patients have specific treatment for COVID-19 with convalescent plasm or remdesivir/oseltamivir, just one patient received hydroxychloroquine, and 12 patients received only symptomatic treatment. The median time between COVID-19 to LT was 19 days (13.5‒44.5).

Regarding immunosuppression, tacrolimus and steroids were administrated in all patients, and 13 had concomitantly used mycophenolate mofetil. The mean follow-up was 64 days (45.5‒101.5). All 13 articles included in the analysis of patients infected by SARS-CoV-2 and posteriorly undergoing LT were case reports/correspondences/case series^10^, ^11^, ^12^, ^13^, ^14^, ^15^, ^16^, ^17^, ^18^, ^19^, ^20^, ^21^, ^22^ (Table 1).

ALF×non−ALF(CLD)

Ten patients with Chronic Liver Disease (CLD) and eight patients with Acute Liver Failure (ALF). There was no difference among groups regarding age, gender distribution, the severity of COVID-19 symptoms, LT modality, and time between COVID-19 and LT (Table 2).

**Table 2:** Comparison between patients with Acute liver failure and Chronic liver disease.

	ALF	CLD	p-value
**Cases (n)**	8	10	-
**Age (years)**	24.0 ±14.1	42.9 ±12.2	0.118
**Men, n (%)**	3 (37.5%)	8 (80%)	0.115
**Time after COVID to LT (days)**	16 (9‒17)	34 (19.5‒52.25)	0.096
**COVID severity (mild)**	6 (75%)	8 (80%)	0.999
**LT modality (DDLT/LDLT)**	5/3	6/4	0.999

ALF, Acute Liver Failure; CLD, Chronic Liver Disease; COVID, Coronavirus Disease; LT, Liver Transplantation; DDLT, Deceased Donor Liver Transplantation; LDLT, Living Donor Liver Transplantation.

## Discussion

The COVID-19 pandemic has strongly affected many lives worldwide and the transplant community, including transplant recipients who urgently need organs. Since the pandemic started, intensive care unit beds have been scarce, thus reducing the odds of procuring transplantable solid organs from suitable donors. Consequently, patients on the waiting list are less likely to be transplanted in due time and more prone to develop life-threatening disease complications.^23^ Systematic reviews have been reported about COVID-19 infection in solid organ transplant recipients.^24^ However, further uncertainty regarding the safety of performing solid organ transplantation in a recipient who has recently recovered from COVID-19. The current knowledge about the latency of SARS-CoV-2 is limited. On the other hand, once transplant recipients develop COVID-19, they are at a higher risk of dying and/or developing end-organ dysfunction.^24^^,^^25^

Despite the concerns regarding the postoperative evolution, the mortality of patients with high MELD or ALF transplanted shortly after COVID-19 diagnosis does not seem to be higher. Interestingly, whilst patients with CLD but without cirrhosis appear to have a similar risk of mortality following SARS-CoV-2 infection compared to patients without liver disease, patients with cirrhosis have an elevated risk of COVID-19 mortality, which may reach 32%.^26^

The median time from COVID-19 diagnosis to LT diagnosis was 19 days in the present report. Most of the studies focused on patients with high MELD and acute-on-chronic liver disease patients. Despite being a small number, the authors identified 3 cases in which the recipient acquired SARS-CoV-2 infection shortly before the LT, and the authors found no significant difference in their initial presentation, clinical course, and outcome when compared to patients who had negative SARS-CoV-2 RT-PCR assay at the moment of LT.^12^^,^^21^^,^^22^ On the other hand, the group has previously shown that COVID-19 diagnosis shortly after LT may be associated with worse outcomes, especially in older patients with comorbidities who acquired the infection during the posttransplant hospitalization period.^27^^,^^28^ Therefore, multimodal strategies are necessary to prevent such infections during the COVID-19 era.^29^

A single COVID-19 survivor died after LT. He was a 41 years-old male who had COVID-19 15 days before LDLT. He had mild symptoms, non-detectable COVID antibodies, and was treated with remdesivir. He developed a postoperative biliary leak, which evolved to sepsis and graft dysfunction.^14^ No respiratory complications in the LT postoperative period were observed. This data demonstrates that patients who recovered from a previous SARS-CoV-2 infection have a similar outcome when undergoing LT compared to those who never developed COVID-19. These findings lead us to believe that previous SARS-CoV-2 infection does not seem to be correlated with a worse post-LT outcome, especially in asymptomatic recipients and/or in those with the interval between COVID-19 diagnosis and LT of at least 19 days.

Mycophenolate Mofetil (MMF) is an independent predictor of severe COVID-19.^30^ MMF and SARS-CoV-2 are associated with worse outcomes,^31^^,^^32^ and this is explained by the cytostatic effect on activated lymphocytes resulting in lymphopenia.^33^ In these studies, immunosuppression was widely debated, 11 recipients got MMF in their immunosuppression regimen. As a part of these, two patients had low dose MMF (250 mg twice daily initially until 1g twice daily),^14^^,^^17^ the remaining started with a standard dosage of 1g twice daily. Therefore, in the authors’ opinion, MMF should be withdrawn if a liver transplant patient presents with SARS-CoV-2 infection at any stage or severity of the disease. However, in recipients who had COVID in the past, the MMF contraindication in the immunosuppression regimen is unclear.

Even though the present study provides a general overview of recipients who recovered from COVID-19 and then underwent LT, it has several limitations. There was no randomized clinical trial, and the number of articles was low; at least 40% of them were short communications (letter to the editor and editor correspondence). Thus, information regarding the clinical status of recipients at the time of LT (such as MELD, presence of COVID-19 antibodies, postoperative evolution, and time of follow-up) was scarce. Moreover, reports with unfavorable outcomes were lacking, maybe due to publication bias (only one recipient's mortality was reported).

In conclusion, despite the concerns regarding the postoperative evolution, the mortality of patients with high MELD or fulminant hepatitis transplanted shortly after COVID-19 diagnosis does not seem to be higher.

## Authors' contributions

All authors have approved the final draft of the manuscript submitted. Study conception and design, data collection and analysis, interpretation, writing the manuscript and literature Search: Nacif LS. Study conception and design, data collection and analysis, interpretation and critical revision: Fernandes MR, Waisberg DR, Pinheiro RS. Study conception, interpretation and critical revision: Rocha-Santos V, Galvão F. Study conception, interpretation and critical revision: Andraus W and Carneiro-D'Albuquerque L.

## Conflicts of interest

The authors declare no conflicts of interest.
